# Traumatic axonal injury in the mouse is accompanied by a dynamic inflammatory response, astroglial reactivity and complex behavioral changes

**DOI:** 10.1186/1742-2094-10-44

**Published:** 2013-04-04

**Authors:** Sara Ekmark-Lewén, Johanna Flygt, Olivia Kiwanuka, Bengt J Meyerson, Anders Lewén, Lars Hillered, Niklas Marklund

**Affiliations:** 1 Department of Neuroscience, Division of Neurosurgery, Uppsala University, Uppsala, 751 85, Sweden; 2 Department of Neuroscience, Division of Pharmacology, Biomedical Center, Uppsala University, Uppsala, 715 23, Sweden

**Keywords:** Astrocytes, β-amyloid precursor protein (β-APP), Central fluid percussion injury, Diffuse traumatic axonal injury, Inflammatory response, Functional outcome, Mouse

## Abstract

**Background:**

Diffuse traumatic axonal injury (TAI), a common consequence of traumatic brain injury, is associated with high morbidity and mortality. Inflammatory processes may play an important role in the pathophysiology of TAI. In the central fluid percussion injury (cFPI) TAI model in mice, the neuroinflammatory and astroglial response and behavioral changes are unknown.

**Methods:**

Twenty cFPI-injured and nine sham-injured mice were used, and the neuroinflammatory and astroglial response was evaluated by immunohistochemistry at 1, 3 and 7 days post-injury. The multivariate concentric square field test (MCSF) was used to compare complex behavioral changes in mice subjected to cFPI (n = 16) or sham injury (n = 10). Data was analyzed using non-parametric statistics and principal component analysis (MCSF data).

**Results:**

At all post-injury time points, β-amyloid precursor protein (β-APP) immunoreactivity revealed widespread bilateral axonal injury and IgG immunostaining showed increased blood–brain barrier permeability. Using vimentin and glial fibrillary acidic protein (GFAP) immunohistochemistry, glial cell reactivity was observed in cortical regions and important white matter tracts peaking at three days post-injury. Only vimentin was increased post-injury in the internal capsule and only GFAP in the thalamus. Compared to sham-injured controls, an increased number of activated microglia (MAC-2), infiltrating neutrophils (GR-1) and T-cells (CD3) appearing one day after TAI (*P*<0.05 for all cell types) was observed in subcortical white matter. In the MCSF, the behavioral patterns including general activity and exploratory behavior differed between cFPI mice and sham-injured controls.

**Conclusions:**

Traumatic axonal injury TAI resulted in marked bilateral astroglial and neuroinflammatory responses and complex behavioral changes. The cFPI model in mice appears suitable for the study of injury mechanisms, including neuroinflammation, and the development of treatments targeting TAI.

## Background

Diffuse axonal injury (DAI) results from rapid acceleration/deceleration or rotation of the brain, subjecting axons to mechanical shearing and stretch forces. DAI is frequently observed following traffic accidents, sports injuries and falls, and across the entire spectrum of traumatic brain injury (TBI) severity [1-4]. The morbidity and mortality associated with TBI are closely linked to the extent of axonal injury in subcortical, central and brainstem white matter tracts. A major consequence of DAI is unconsciousness and persistent vegetative state that can be observed up to many years following human TBI [2,5-11]. Axonal damage has also recently been recognized as a key predictor of outcome in other human central nervous system (CNS) diseases including spinal cord injury, metabolic encephalopathy and multiple sclerosis [12]. Unfortunately, spontaneous axonal regeneration is strictly limited following axonal injury in the CNS. Although several pharmacological compounds such as antibodies targeting Nogo-A and/or its receptors have shown promising preclinical efficacy in axonal injury models, there are currently no treatment options with proven clinical efficacy available for DAI patients [13-15].

On the cellular level, axonal injuries can be morphologically characterized by antibodies targeting the amyloid precursor protein (β-APP) identifying impaired axonal transport [16-21]. Accumulation of β-APP is a hallmark finding of DAI, occurring either as periodic localized swellings, axonal varicosities or as a single large axonal swelling, the classical axonal bulb [22]. In order to understand the complex pathophysiology of DAI and to develop novel treatments, clinically-relevant animal models of traumatic axonal injury (TAI, the experimental counterpart of DAI) are needed. The central fluid percussion injury (cFPI) [23] and the impact acceleration injury model [24] are both useful models for studying TAI in the rat with features mimicking human DAI. Mouse models of TBI have several advantages in the study of cellular and molecular injury responses and recently, the cFPI model of TAI was adopted for the mouse [5]. The neuroinflammatory response to TAI was previously studied using cFPI in the rat [25] and closed head injury in mice [26] where long-term microglial activation was observed. To our knowledge, glial cell reactivity, inflammatory responses and behavioral outcome have not previously been studied in the novel mouse cFPI TAI model. In the present study, mice subjected to a moderate cFPI were followed for up to one week post-injury where glial cell reactivity and the inflammatory responses were evaluated using immunohistochemistry.

Since behavioral disturbance is a common clinical problem, behavioral evaluation was used and we hypothesized that TAI produces unique behavioral changes. Thus, the functional outcome was analyzed at 2 to 9 days post-injury using the multivariate concentric square field (MCSF) test comparing complex behavioral patterns of mice subjected to cFPI to those of sham-injured controls [27,28]. We show that the cFPI model causes a dynamic and widespread, bilateral astroglial and neuroinflammatory response in addition to axonal injury in important white matter tracts. The astroglial response was more widespread than the axonal injury and neuroinflammatory response. Compared to sham-injured controls, mice subjected to cFPI had different behavioral patterns. The cFPI model also appeared to induce unique behavioral deficits, suggested by comparing previously published behavioral MCSF data obtained from mice subjected to a widely used focal contusion TBI model [28]. Thus, the cFPI model in mice appears highly suitable for the study of TAI-related injury mechanisms and our results lead us to hypothesizethat glial reactivity and neuroinflammation play important roles in the pathophysiology of axonal injury following TBI.

## Methods

### Animal care and housing

Male adult C57BL/6 mice (Taconic, Denmark) housed in groups of six per cage with food and water *ad libitum* on a 12-h light/dark cycle with lights on at 7.00 AM were used in the study. All animals remained in the animal care facility for a minimum of seven days prior to the experiments. Twenty-nine mice (weight 21 to 28 g) were used for the immunohistochemical study and 30 mice (weight 22 to 29 g) for the functional outcome evaluation. Behavioral testing was performed during the light period of the cycle. To reduce stress, animals were handled for one week prior to the behavioral testing. Handling procedures included daily transfer from the home cage and placement of the mouse on the arm of the scientist for 1 to 2 min after which it was returned to its home cage. The same scientist (S.E-L) conducted the surgical procedures and functional outcome studies. All experiments were approved by the Uppsala County Animal Ethics board, and followed the rules and regulations of the Swedish Animal Welfare Agency. All analyses were performed by investigators blinded to the injury status and survival time point of each animal.

### Central fluid percussion injury (cFPI)

For the immunohistochemical evaluation, mice were randomly subjected to either cFPI (n = 20) or sham injury (n = 9). Three animals died immediately following the injury due to prolonged apnea and two animals were excluded from the study due to a dural tear. In total, the study included five brain-injured and three sham-injured mice per time point, allowed to survive 1, 3 or 7 days post-injury. For the functional outcome evaluation, 20 animals were subjected to cFPI. Four animals died immediately post-injury and 16 mice were evaluated. The exclusion criteria for apnea duration were set to a maximum of 60 seconds [29,30]. Sham-injured animals (n = 10) were subjected to anesthesia and surgical preparation including craniotomy but did not receive the pressure pulse.

The cFPI surgical procedure was modified from those previously described by Dixon *et al.*[27] in rats and by Greer *et al.*[5] in mice. Anesthesia was induced in a chamber with 4% isoflurane in air. The mice were moved to a stereotaxic frame and a mixture of isoflurane (1.2 to 1.4%) and N_2_O/O_2_ (70/30%) was delivered through a nose cone to maintain general anesthesia under spontaneous breathing. Body temperature was maintained at 37°C by a heating pad coupled to a rectal probe (CMA 150, CMA Microdialysis AB, Solna, Sweden) aided by an overhead-heating lamp. After shaving and cleansing the skin with ethanol, bupivacaine 2.5 mg/mL (Marcaine®, AstraZeneca, Sweden) was subcutaneously applied and the scalp was opened by a midline incision. Artificial tear lubricant (Viscotears, Novartis, Inc., Basel, Switzerland) was used for corneal protection during anesthesia.

**Figure 1:**
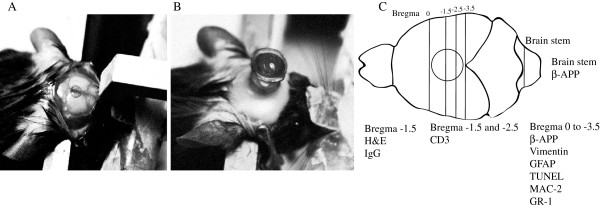
**Central fluid percussion injury (cFPI) procedure. **(**A**) To prepare for cFPI, a 3.0 mm diameter craniotomy was made in the midline between the bregma and lambda sutures, leaving the underlying dura intact; (**B**) Using dental cement, a cap filled with normal saline was sealed over the craniotomy; (**C**) Overview of the bregma levels where the different immunohistochemical analyses were prepared.

A 3.0 mm diameter craniotomy was made in the midline between the bregma and lambda sutures, leaving the underlying dura intact. Hemorrhages, if present, were controlled by gentle pressure on the bone edge with a Q-tip. The bone flap was placed subcutaneously during the preparation for cFPI. A plastic cap was secured over the craniotomy using dental cement (HeraeusKulzer GmbH, Hanau, Germany), and the integrity of the seal between the cap and the skull was confirmed by adding normal saline into the cap (Figure 1 A,B). Injury was produced by attaching the saline-filled hub to the Luer-Lok fitting on the fluid percussion device (VCU Biomedical Engineering Facility, Richmond, VA, USA) and releasing a pendulum striking the end of a saline-filled reservoir transmitting a pressure wave into the closed cranial cavity. The pressure pulse measured by the transducer was displayed on an oscilloscope and the peak pressure was recorded in atmospheres (atm). Immediately after the injury, the animal was inspected for apnea and seizures defined as fore- and/or hind-limb twitching. Following resumption of spontaneous breathing, the mouse was re-anesthetized with isoflurane and the cement and cap were removed, the bone flap was dried and replaced over the craniotomy, and the skin was closed with sutures. Animals were moved to a cage placed under a warming lamp until recovered from anesthesia and fully ambulatory. As a measure of animal health, the weight of the animal was monitored daily for a minimum of three days following injury, or until they showed weight gain. Weight-loss more than 20% was an exclusion criterion, mandated by the animal ethics committee.

### Morphological methods

#### Immunostaining

At time for sacrifice, mice were given an overdose of isoflurane and were transcardially perfused with 4% formaldehyde. The brains were cryoprotected in 30% sucrose and snap frozen in cold isopentane, and thereafter cryosectioned (HM500, Microm GmbH, Walldorf, Germany). Fourteen μm thick coronal sections from bregma levels 0, -1.5, -2.5 and −3.5 mm [31] were analyzed (Figure 1C). On each glass, three consecutive sections were mounted. Additionally, the brain stems were cut sagitally.

For general morphology, sections from level −1.5 mm bregma were fixed in acetone, rehydrated in alcohol (absolute ethanol to 80% ethanol) and washed in tap water. Nuclei were stained with Mayer’s hematoxylin for 2 min, thereafter rinsed in tap water, followed by eosin staining for a few seconds and dehydration in alcohol (80% to absolute ethanol and xylene), and finally mounted.

Sections prepared for immunohistochemistry were fixed in cold acetone, washed in phosphate buffered saline (PBS) and treated in pre-heated citrate buffer for 5 min. They were then washed in PBS and incubated in peroxidase blocking solution (DakoCytomation A/S, Glostrup, Denmark) for 20 min, washed and blocked with 10% serum (horse or goat serum depending on the primary antibody) in PBS with 0.3% triton-X. The primary antibodies were used over night at 4°C (Table 1). Sections stained for blood–brain barrier (BBB) leakage were not incubated with a primary antibody; instead, only a biotinylated anti-mouse IgG secondary antibody was used at 1:100 for one hour in room temperature (Vector Laboratories, Burlingame, CA, USA). Sections were thereafter washed with PBS and incubated for 60 min with a secondary biotinylated antibody (Table 1). Following washing with PBS, sections were incubated for 30 min with avidin-biotin-peroxidase complex (Vector Laboratories) at a dilution of 1:200. Staining was developed using 3’,3’-diamionbenzidine (DAB, Vector Laboratories) as chromogen and was counterstained with hematoxylin. To intensify the reaction product of β-APP and CD3, the nickel enhancement procedure was applied. Negative controls included omission of the primary antibody. As a positive control for CD3 staining, mouse spleen was used. Staining for terminal deoxynucleotidyltransferasedUTP nick end labeling (TUNEL) included incubation with citrate buffer for 60 min in 90°C, incubation with 5% normal goat serum and 0.3% Triton-X in PBS and incubation with TUNEL mix (Roche Diagnostics GmbH, Mannheim, Germany) for 60 min at room temperature. Sections were then washed in PBS and mounted using Vectashield with DAPI (Vector Laboratories).

**Table 1:** Overview of the primary and secondary antibodies

**Antibody**	**Host animal/concentration**	**Target**	**Supplier**
**β-APP**	Rabbit, 1:200	Amyloid precursor protein	Invitrogen Life Technologies Europe, BV, Netherlands
**Vimentin**	Goat, 1:200	Astrocytes	Santa Cruz Biotechnology, Inc. CA, USA
**GFAP**	Rabbit, 1:500	Astrocytes	Dakocytomation A/S Glostrup, Denmark
**MAC-2**	Rat, 1:200	Microglia	Cederlane, Ontario, Canada
**GR-1**	Rat, 1:200	Neutrophils	Biolegend, San Diego, USA
**CD3**	Hamster, 1:100	T-Cells	AbSerotec, Dusseldorf, Germany
**Biotinylated anti-mouse IgG**	Mouse, 1:100	Blood brain barrier leakage	Vector laboratories, Burlingame, CA, USA
**Biotinylated anti-rabbit IgG**	Rabbit, 1:200	Secondary antibody	Vector laboratories
**Biotinylated anti-goat IgG**	Goat, 1:200	Secondary antibody	Vector laboratories
**Biotinylated anti-rat IgG**	Rat, 1:200	Secondary antibody	Vector laboratories
**Biotinylated anti-hamster IgG**	Hamster, 1:200	Secondary antibody	Vector laboratories

#### Microscope analysis and cell quantification

**Figure 2:**
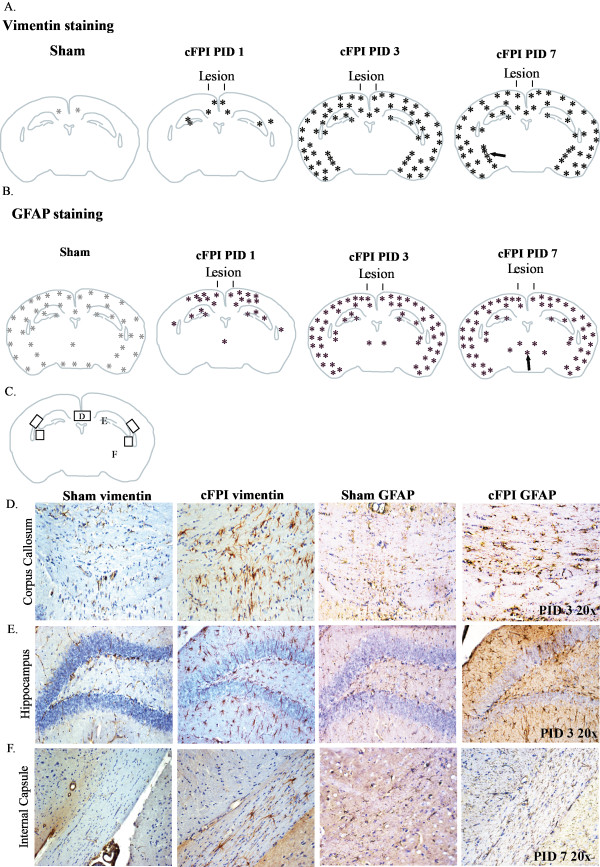
**Schematic overview of the vimentin and GFAP staining after central fluid percussion injury (cFPI) in mice at post-injury day 1,3 and 7.** (**A**) The immunoreactivity for vimentin was increased in the cortex, the subcortical white matter, the fimbria of hippocampus and the dentate gyrus of hippocampus in addition to the external and internal capsule (arrow), especially at 3 days post-injury. Note the absence of increased vimentin staining in the thalamus; (**B**) Sham-injured animals showed some background staining of GFAP, which increased after cFPI. The increased GFAP staining was observed in the cortex, the subcortical white matter, the fimbria of hippocampus and the dentate gyrus of the hippocampus and in the thalamus (arrow). Note the absence of increased GFAP staining in the internal capsule; (**C**) Vimentin and GFAP staining in three different brain regions after cFPI or sham injury; (**D**) The corpus callosum; (**E**) the dentate gyrus of the hippocampus and (**F**) the internal capsule. PID: Post-injury day.

Sections were analyzed in the microscope (Zeiss Axiovision, Zeiss Gmbh, Göttingen, Germany) and immunohistochemical images were captured for cell counting of Gr-1 and Mac-2. Using Axiovision image analysis software (Zeiss Axiovision), images for quantification of immune cells were analyzed from bregma levels 0; -1.5; -2.5 and −3.5 mm ([31], two to three sections per animal) in the following white matter tracts; corpus callosum, external capsule and fimbria of hippocampus. CD3-positive cells were manually and exhaustively counted at 20× magnification directly in the microscope at the level of bregma −1.5 and −2.5 mm (one section per bregma level). A grid of 700×500 μm was placed in the region of interest in all evaluated white matter tracts (the corpus callosum, external capsule and the fimbriae; Figure 2C). The total number of cells per animal was used and the mean number of cells per group is presented. For hematoxylin and eosin staining and BBB staining, bregma level −1.5 mm (three sections per animal) was analyzed for all animals. β-APP, vimentin, GFAP and TUNEL staining was analyzed at all levels (bregma level 0, -1.5, -2.5 and −3.5, two to three sections per level). To test the hypothesis that the cFPI model in mice also produces brain stem axonal injury, three sagittal sections from the brain stem (Figure 1C) of each animal were descriptively evaluated for β-APP staining. Analysis of the area stained with IgG (in mm^2^) was performed using a computer-based image analysis system (Image J 1.45, Wayne Rasband, National Institutes of Health, Bethesda, USA). A threshold for positive IgG staining was chosen by manually evaluating a control (sham) animal for IgG staining and this threshold was then kept equal for all included animals. The stained area was then converted to mm^2^ using a reference area.

### Functional outcome evaluation

**Figure 3:**
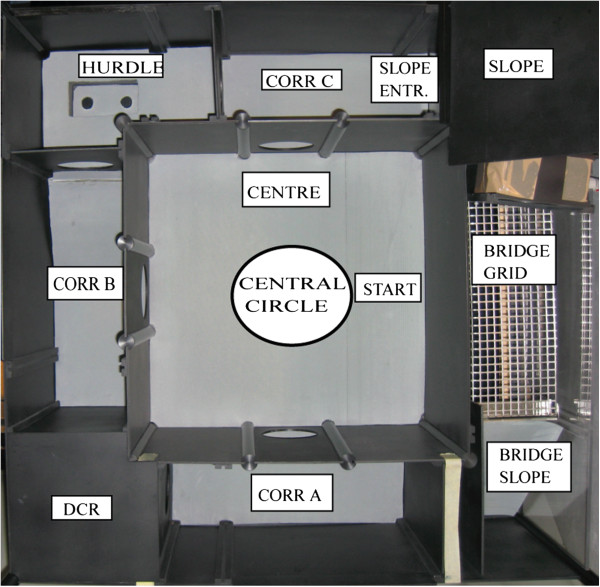
**The Multivariate Concentric Square Field (MCSF) arena and the defined zones.** CORR **A**, **B** and **C**, indicate corridors **A-C**; DCR is the dark corner room; and SLOPE ENTR is the slope entrance. START indicates where the animal is placed when introduced to the arena. Picture modified from [28].

To study potential differences in behavioral patterns between cFPI mice and sham-injured mice, we used the multivariate concentric square field (MCSF) test using the same variables as previously described in detail [28]. The MCSF provides several areas for the animal to explore by free choice and includes sheltered, open and elevated areas, a hole-board device and areas with different lighting. The entire arena was divided into zones (Figure 3) that formed the basis of the description and the variables of the animal’s performance in the test. Each mouse subjected to cFPI or sham injury was tested alone in the MCSF at post-injury day 2 (trial 1) and 9 (trial 2). The testing session lasted for 20 min and the arena was cleaned between each test. Behaviors measured in the MCSF were frequencies (FRQ) to the different zones, duration (DUR, sec) in different zones, latencies (LAT, sec) to first visit in different zones and for some variables duration/visit in zones. The variable “total activity” is the sum of all visits to the different zones, “total corridor”is the sum of corridors A-C, distance moved (cm) in the whole arena. Velocity (cm/sec) was also measured.

### Behavioral recordings

The animals were monitored by a TV-video set-up (Panasonic Super Dynamic Wv- BP550/B camera and Panasonic NV-HD640 VHS recorder; Panasonic, Stockholm, Sweden). The manual scoring of visits to different zones, FRQ, LAT, DUR and recording of rearing and grooming was performed using the software Score version 2.0 (Pär Nyström, Copyright Solids, Uppsala, Sweden). The Ethovision software version 2.3 (Noldus Information Technology, Wageningen, Netherlands) was used for recordings of velocity (cm/sec) and distance moved (cm) in the MCSF arena.

### Statistical analysis

Cell counts and behavioral data were found not to meet the assumption of normal distribution using the Shapiro-Wilk’s *W-*test. Thus, Kruskal-Wallis ANOVA was used and was followed by Mann–Whitney *U*-test for pair-wise comparisons. After positive test for normal distribution, areas stained for IgG where analyzed with one-way ANOVA and Tukey post-hoc test. Parametric data is presented as means ± standard deviations. A *P* value <0.05 was considered statistically significant. All analyses were performed using Statistica 10.0 software (StatSoft Inc., Tulsa, OK, USA).

For the behavioral analysis, principal component analysis (PCA, [32]) was used as a complement to traditional statistics, to create a score plot showing a summary of the relationship among the individuals and a loading plot in which variables important to these relationships can be identified. The two plots are complementary and superimposable. This statistical method is very useful for analysis of material with large numbers of variables in few numbers of animals [33,34]. SIMCA-P + 12 software version 12.0 (Umetrics AB, Umeå, Sweden) was used for this purpose.

## Results

The cFPI device created a pressure pulse of 1.50 ± 0.24 atm transmitted into the closed cranial cavity of the animal. In all brain-injured mice, the injury resulted in a 35 ± 18 s (range 17 to 59 s) long apnea. A short-lasting seizure, typically <5 s, immediately after the impact was also observed in the forelimbs and/or hind legs in all but two animals. The acute mortality rate was 17.5% caused by long-lasting apnea (>60 s).

### General morphology and blood–brain barrier leakage

On macroscopic inspection, no sham-injured mice showed traumatic subarachnoid blood on the brain surface. At one day after sham injury, one third of animals had a few, minor superficial cortical hemorrhages. In contrast, cFPI mice typically had traumatic subarachnoid blood on the brain surface at 1 and 3 days post-injury and superficial cortical hemorrhages were also observed in the parietal cortex in the majority of animals at these time points (data not shown).

**Figure 4:**
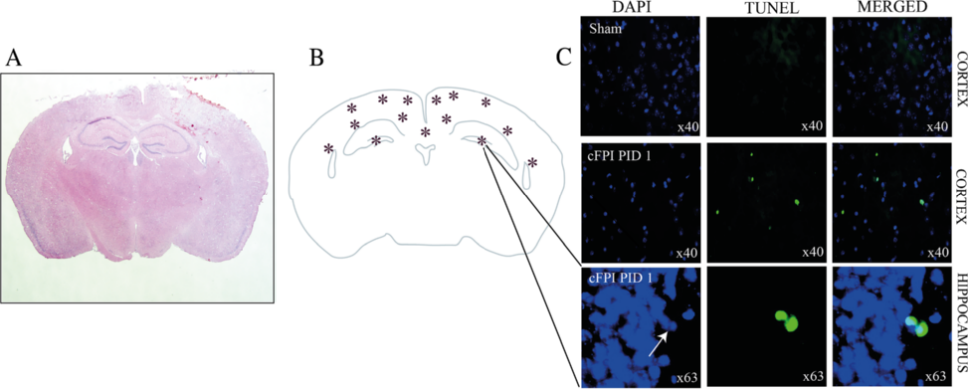
**Post-injury hemorrhages and apoptosis.** (**A**) Overview of the injured brain at bregma level −1.5 mm (see Figure 1C) at one day after cFPI, a model of TAI in mice. The injury produces small hemorrhages in the cortex underlying the impact; (**B**) Overview of the TUNEL staining distribution at one day post-injury; (**C**) Immunohistochemical image shows apoptotic cells in the cortex and dentate gyrus of the hippocampus although not in sham-injured controls. PID: Post-injury day.

On H&E-stained coronal sections, two sham-injured mice showed minimal, superficial damage to the cortical surface, although a deep lesion and/or a cortical cavity were not observed. In brain-injured animals, small hemorrhages were present in the cortical region underlying the impact and in the subcortical white matter (Figure 4A). The cortical injury did not extend into the deeper layers of the cortex and did not result in a large cortical cavity. At seven days post-injury, only a few hemorrhages remained in the cortex. TUNEL staining was undertaken to study cell death at one day post-injury. There were frequent cells undergoing apoptotic cell death in the cortex underlying the impact (Figure 4B,C) but only a few TUNEL-positive cells were observed in the subcortical white matter and in the hippocampus (Figure 4C). In 3/5 cFPI animals, there was intraventricularhemorrhage at one day post-injury (data not shown).

**Figure 5:**
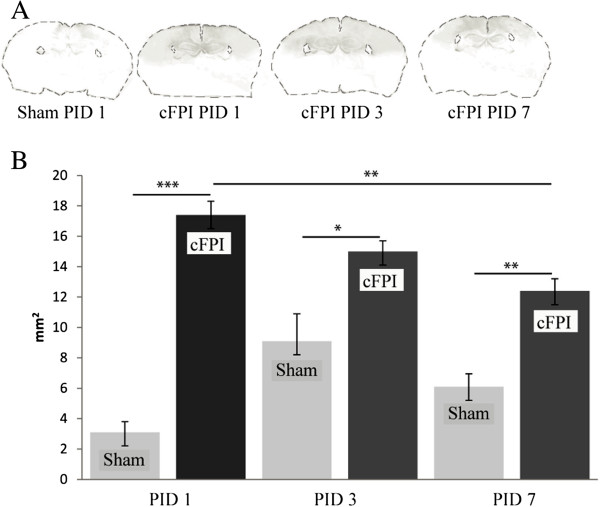
**Blood-brain barrier leakage evaluated by mouse IgG staining.** (**A**) Representative images of blood–brain barrier leakage of sham- and brain-injured animals, identified by IgG staining in the brain regions underlying the craniotomy and impact site; (**B**) Quantitation of the area (mm^2^) stained with IgG (above the pre-set intensity level) at 1, 3 and 7 days after sham injury or central fluid percussion injury (cFPI), presented as means ± SD. The IgG staining was more intense in sham-injured controls at 3 days compared the 1 and 7 days post-injury time points, although without reaching statistical significance. *P<0.05; **P<0.01; ***P<0.001 compared to sham-injured controls. PID: Post-injury day.

In sham-injured animals, some IgG staining was apparent in the cortex. Although the staining intensity was highest at three days post-injury, there were no significant differences between the post-injury days. The staining of mouse IgG in the brain regions, including the neocortical and hippocampal areas, underlying the impact had increased 5.7 ± 1.9 fold (560%) at one day post-injury, indicating increased BBB permeability in these regions (*P*<0.05 compared to sham-injured controls; Figure 5A,B). Although a 2.0 ±1.2 fold (200%) increase in BBB permeability compared to sham-injured controls was observed up to 7 days post-injury, the staining became progressively weaker with time (*P*<0.01 between 1 and 7 days post-injury).

### cFPI produces axonal injury identified by β-APP immunoreactivity

**Figure 6:**
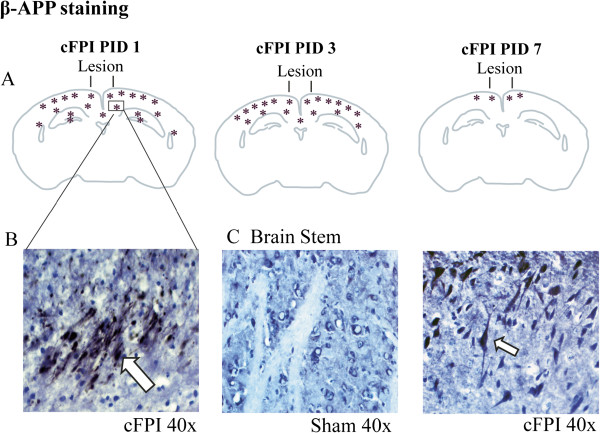
**Axonal injury after cFPI in mice shown by β-APP immunostaining.** (**A**) Schematic distribution of the β-APP staining in the various brain regions at 1, 3 and 7 days post-injury; (**B**) Axonal profiles stained with β-APP in the corpus callosum at 1 day post-injury (for example see arrow); (**C**) β-APP positive axonal profiles in the brain stem at 1 day post-injury (right; for example see arrow), compared to a sham-injured animal with normal staining of nerve cell bodies (left). PID: Post-injury day.

In sham-injured animals, weak β-APP immunoreactivity was observed in the cortex and brain stem (Figure 6C left) within nerve cell bodies whereas dendrites, axons and astroglial processes were unstained.

One day after cFPI, immunoreactive β-APP-positive axonal profiles appeared in subcortical white matter (Figure 6B). A few β-APP-positive axonal profiles were also observed in the dorsolateral rostral brain stem at 1 day post-injury (Figure 6C), although not at later post-injury time points. A few β-APP-positive axons were also observed in the dentate gyrus of the hippocampus. The β-APP immunoreactivity peaked at 1 day, declined at 3 days and at 7 days post-injury only a few β-APP immunoreactive cells remained in the superficial cortex underlying the impact site (Figure 6A).

### Vimentin and GFAP immunoreactivity distribution differs after cFPI

In order to determine the astroglial response after cFPI, analysis of vimentin and GFAP immunostaining was performed. The brains of sham-injured control animals displayed vimentin immunoreactivity only in ependymal cells and to a minimal extent in the neocortex (Figure 2A).

One day after cFPI, there were star-shaped vimentin positive cells in the subcortical white matter and a few cells appeared in the hippocampal CA2 region (Figure 2A). At 3 days post-injury there were numerous immunoreactive cells widespread in the cortex, in the subcortical white matter including the corpus callosum and external capsule, and in the hippocampus (Figure 2). There were also vimentin-positive cells lining the lateral ventricles (different from the ependymal cells observed in sham-injured controls) and in the internal capsule although not in the thalamus (Figure 2A). Although the expression of vimentin persisted in the thalamus at 7 days post-injury, there were markedly fewer cells immunoreactive for vimentin in the hippocampus. In the cortex, there were fewer vimentin-positive cells in contrast to the internal capsule, showing more vimentin-positive cells at this time-point (Figure 2A).

Immunostaining of GFAP was observed in sham-injured controls in the white matter, in the hippocampus and to some extent in the neocortex and in the ependymal cells (for an overview see Figure 2B). There was a slightly increased GFAP staining intensity in the cortex and the subcortical white matter in 2/3 of the sham-injured animals after 1 and 3 days post-injury at bregma levels 0 and −1.5 mm compared to other brain regions.

At 1 day post-injury, the GFAP immunoreactivity was increased in the cortex, in the hippocampus and to some extent in the external capsule compared to sham-injured controls. By 3 days post-injury, there were numerous star-shaped GFAP-positive astrocytes throughout the neocortex, in the hippocampus and external capsule (Figure 2B,E). The GFAP immunoreactivity was not increased in the internal capsule (Figure 2B,F). A few GFAP-positive cells also appeared in the thalamus. At 7 days post-injury, the number of GFAP-positive astrocytes in the thalamus had increased. Overall, the immunoreactivity of GFAP in the hippocampus was weaker compared to 3 days post-injury (Figure 2B).

### The neuroinflammatory response

**Figure 7:**
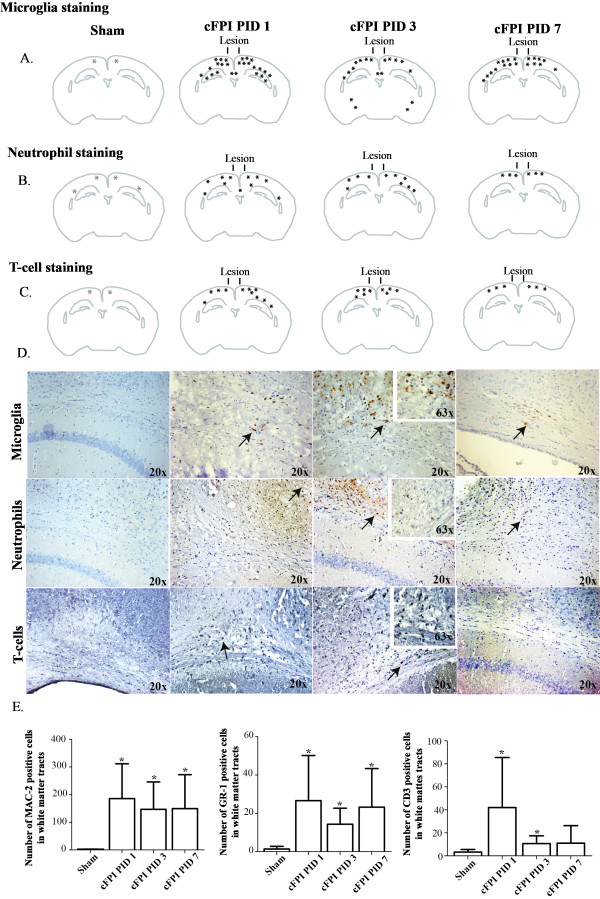
**Overview of the distribution of microglial, neutrophil and T-cell staining at 1, 3 and 7 days after cFPI or sham-injury in mice.**(**A-C**) The microglial staining was increased throughout the observation period in the cortex and in the subcortical white matter, especially at one day post-injury. Infiltrating neutrophils appeared at one day post-injury in the cortex and subcortical white matter. T-cells were observed in the cortex and in the subcortical white matter after one day post-injury but then the number of cells declined; (**D**) The inflammatory response after cFPI or sham-injury. The images show activated microglia/macrophages (MAC-2), neutrophils (GR-1) and T-cells (CD3) in the external capsule from post-injury day (PID) 1 to 7, examples of cells staining positive for the cell type marker are indicated with arrows. Inserts show the same area in the external capsule at a higher magnification; (**E**) Cell counts of microglia/macrophages, neutrophils and T-cells in the corpus callosum, external capsule and fimbria of hippocampus from 1, 3 and 7 days post-injury (presented as means ± SD). *Indicates a statistically significant difference compared to sham-injured controls (*P*<0.05).

To establish the time-course of immune cell infiltration into the cortex and white matter in the cFPI model, the number of three different immune cell types was estimated in the corpus callosum, the external capsule and the fimbriae (Figure 7A-C).

Sham-injured controls showed very few or no cells that were immunoreactive for Mac-2 (activated microglia/macrophages), GR-1 (neutrophils) or CD3 (T-cells; Figure 7A-E). One day post-injury, numerous activated microglia/macrophages were shown in the cortex, in white matter tracts including the corpus callosum, the external capsule and the fimbriae, and in the hippocampus (Figure 7A,D). The number of Mac-2 positive cells was elevated compared to sham-injured controls (*P*<0.05) without significant changes from 1 to 7 days post-injury (Figure 7E). These cells were observed in healthy-appearing tissue, *i.e.*, without signs of cell death or necrosis. Neutrophils, stained with GR-1, were observed in the same regions as the microglia/macrophages although the number of cells was lower. The number of cells was unchanged over time with only a minimal decrease at 3 days post-injury (Figure 7B,E). The number of CD3-positive T-cells peaked at 1 day post-injury and then declined (Figure 7C-E). Infiltrating neutrophils and T-cells were predominately visualized in injured cortical and subcortical regions underlying the impact (Figure 7C).

### Functional outcome evaluation

In both trial 1 and trial 2 of the MCSF test, some significant differences between sham-injured controls and cFPI animals were observed (Table 2). In general, in the first trial, cFPI mice crossed the centre of the arena less frequently (FRQ CENTRE, *P*<0.05) and made shorter visits to the hurdle (DUR/FRQ HURDLE, *P* <0.05) compared to sham-injured controls. There were no significant differences in distance moved between cFPI mice (5,185 ± 1,074 cm) or sham-injured mice (4907 ± 933 cm). In the second trial, cFPI mice crossed the centre less frequently (FRQ CENTRE, *P*<0.05), had fewer visits to the corridors (TOTAL CORRIDORS, *P*<0.05) and were less active in the whole arena (TOTAL ACTIVITY, *P*<0.05) compared to sham-injured controls. Brain-injured mice spent more time in the centre of the arena (a non-significant trend) and less time in other zones compared to sham-injured controls. By direct observation in the MCSF test, it was clear that cFPI mice were running around in circles in the centre of the arena, indicating a pathological behavior.

**Table 2:** MCSF results following sham injury and cFPI brain injury

**PARAMETERS**	**Sham Trial 1**	**cFPI Trial 1**	**Sham Trial 2**	**cFPI Trial 2**
**LATENCY (sec)**				
LEAVE CENTRE	78.3 ± 50.5	150 ± 285.8	5.2 ± 3.9	12 ± 13.5
DCR	217.9 ± 188	301.1 ± 299.5	109 ± 19.9	73.3 ± 67.5
SLOPE ENTRANCE	164.4 ± 111.5	261.3 ± 287.6	89.5 ± 98.8	76.5 ± 131.4
SLOPE	525.8 ± 425.2	650 ± 390.7	577.9 ± 495.4	627.1 ± 509.2
BRIGDE	639.9 ± 497.6	768.6 ± 421.8	682.1 ± 485.8	689.3 ± 516
HURDLE	179.2 ± 154	267.4 ± 292	58.4 ± 64.5	106.4 ± 98.9
CENTRAL CIRCLE	31 ± 45.5	55.7 ± 53.7	130.1 ± 109.3	87.9 ± 86.9
REARING	59.7 ± 40.4	49.9 ± 35.7	65.8 ± 70.8	34.8 ± 45.3
GROOMING	363.2 ± 347.3	280.7 ± 360.2	570.7 ± 413.9	547.7 ± 495.9
**FREQUENCY**				
CENTRE	24.1 ± 5.7	17.4 ± 7 ^*^	36.6 ± 6.4	29.4 ± 9.4^*^
DCR	10.5 ± 4.4	8.1 ± 4.4	16.1 ± 6.2	16.5 ± 5.8
SLOPE ENTRANCE	8.4 ± 2.0	7.3 ± 3.5	10.5 ± 2.1	10.1 ± 3.6
SLOPE	2.7 ± 1.8	2.1 ± 2	3.5 ± 3.1	2.3 ± 2.3
BRIDGE	3.7 ± 3.5	3.1 ± 3.4	2.7 ± 2.6	2.8 ± 2.8
HURDLE	10 ± 2.9	11.7 ± 5.2	13.1 ± 3	11.1 ± 4.3
CENTRAL CIRCLE	10.5 ± 4.4	13.8 ± 7	8.9 ± 4.2	8.9 ± 4.6
TOTAL CORRIDORS	33.3 ± 6.9	28.1 ± 10.1	58.8 ± 6.4	51.1 ± 12.7 ^*^
REARING	63 ± 23	81.8 ± 20.7	62.5 ± 24	60.1 ± 28.5
GROOMING	1.9 ± 1.1	2.7 ± 1.5	1.2 ± 0.8	0.9 ± 0.9
**DURATION (sec)**				
CENTRE	226.3 ± 53.3	345.2 ± 234	127.7 ± 21.5	139.2±13
DCR	166.6 ± 72.2	149.2 ± 99.2	235.9 ± 90	334.2 ± 160.1
SLOPE ENTRANCE	51.7 ± 10	44.8 ± 23.6	59.5 ± 21.7	57.1 ± 25.7
SLOPE	70.7 ± 50	57.8 ± 57.9	45.3 ± 41.6	50.3 ± 47.7
BRIGDE	56.8 ± 55.7	47.7 ± 47.1	39.6 ± 45.2	42.2 ± 47.5
HURDLE	175.2 ± 61.1	154.6 ± 67.3	178.8 ± 53.8	138.1 ± 59.2
CENTRAL CIRCLE	13.2 ± 20.7	13.9 ± 21.5	4.4 ± 2.6	4.4 ± 2.3
TOTAL CORRIDORS	401.5 ± 68	359.7 ± 120.1	464.3 ± 98.8	399.9 ± 91.7
GROOMING	12.6 ± 9.2	29.5 ± 30.8	9.3 ± 9.9	5.8 ± 7.2
**DURATION/FREQUENCY**				
DCR	16 ± 4.3	17.4 ± 8	15 ± 3.7	20.7 ± 8.5
SLOPE ENTRANCE	6.4 ± 1.7	6.3 ± 3.4	5.7 ± 1.7	5.5 ± 2
SLOPE	20.5 ± 15	23.7 ± 22.3	9.1 ± 8.1	22.6 ± 38.4
BRIDGE	9.4 ± 8.7	11.8 ± 11.1	8.4 ± 7.9	8.8 ± 8.7
HURDLE	17.7 ± 4.8	12.6 ± 4.4 ^*^	13.9 ± 3.6	12.7 ± 3.8
TOTAL CORRIDORS	12.7 ± 3.4	12.6 ± 5.1	8.1 ± 2.4	8.1 ± 2.3
**ACTIVITY MEASURES**				
TOTAL ACTIVITY	105 ± 19.8	95 ± 25.6	157.7 ± 19.3	137.2 ± 31.9 ^*^
DISTANCE MOVED (cm)	4907 ± 933	5185 ± 1074.5	4865 ± 745.7	4644 ± 1204
VELOCITY ARENA (cm/s)	5 ± 0.7	5.5 ± 0.8	5.1 ± 0.7	4.7 ± 1.1
VELOCITY CENTRAL CIRCLE (cm/s)	14.8 ± 4.1	15.6 ± 5.4	22.8 ± 3.7	20.5 ± 8.9
HEAD DIPS	16.5 ± 7.7	9.8 ± 7.4	13 ± 4.6	13.1 ± 8.1

Behavioral parameters of sham-injured and cFPI brain-injured mice in the multivariate concentric square field test during the first and second trial at post-injury day 2 and 9, respectively. Values represent mean ± standard deviation. Sham n = 10, cFPI n = 16. *P<0.05 compared to sham-injured mice analyzed by Kruskal-Wallis test and Mann–Whitney *U* test.

cFPI: Central fluid percussion injury; DCR: Dark corner room.

**Figure 8:**
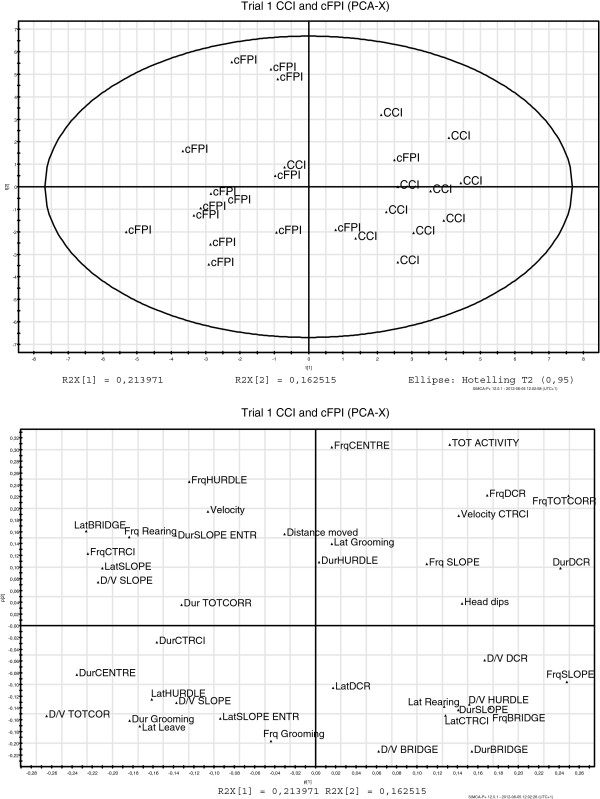
**The principal component analysis (PCA) from the first MCSF test at 2 days post-injury.** To test the hypothesis that the mouse cFPI model induces unique behavioral deficits compared to a focal traumatic brain injury (TBI) model, we extracted historical data [28] obtained under an identical set-up and by the same investigator. (**A**) PCA score plot showing a comparison between mice subjected to the controlled cortical impact (CCI, producing a focal TBI) model and the cFPI model, producing wide-spread traumatic axonal injury. The behavioral patterns of the two TBI models are markedly different; (**B**) PCA loading plot illustrating the MCSF variables that were included in the analysis. Variables located further away from the origin are most important for the model. CCI: Controlled cortical impact, n = 11 (from [28]); cFPI: Central fluid percussion injury, n= 15; CTRCI: Central circle; Dur: Duration; D/V: Duration per visit; Frq: Frequency; Lat: Latency; SLOPE E: Slope entrance; TOT ACTIVITY: Total activity in arena; TOTCORR: Total corridor entries.

**Figure 9:**
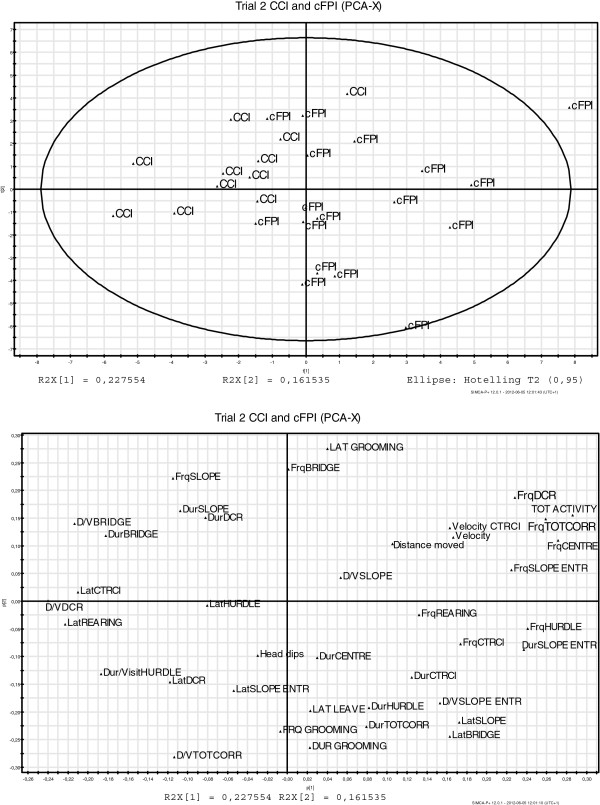
**The principal component analysis (PCA) from the second MCSF test, 7 (CCI-injured animals, **[28]**) or 9 (cFPI animals) days post-injury.** (**A**) PCA score plot showing the locations of the CCI and cFPI groups, indicating different behavioral patterns in the two groups; (**B**) PCA loading plot illustrating the MCSF variables that are included in the analysis. Variables located further away from the origin are most important for the model. CCI: Controlled cortical impact, n = 11 (from [28]); cFPI: Central fluid percussion injury, n = 16; CTRCI: Central circle; Dur: Duration; D/V: Duration per visit; Frq: Frequency; Lat: Latency; SLOPE E: Slope entrance; TOT ACTIVITY: Total activity in arena; TOTCORR: Total corridor entries.

Historical data obtained from a previously published report using the MCSF in mice subjected to the controlled cortical impact TBI model [CCI; 28] allowed us to compare the behavioral deficits caused by diffuse (cFPI) and focal (CCI)TBI. The MCSF data from CCI-injured animals was obtained using the same set-up performed by the same investigator (S. E-L) as in the present study and it was apparent that the behavioral profiles of cFPI mice and CCI-injured mice [28]) were markedly different (Figures 8 and 9).

## Discussion

The aim of the study was to characterize the astroglial reactions, neuroinflammatory response and complex behavioral changes following cFPI in mice, a model of diffuse TAI. In sham-injured controls, there was some BBB leakage, a transient vimentin response and a limited number of infiltrating immune cells, presumably caused by drilling of the thin skull bone. These findings suggest that sham-injured animals are different from naïve animals, as previously shown by us [28,35] and others [26,36]. The cFPI resulted in an acute apnea and seizures with 18% mortality. In the injured brains, there was a long-lasting permeability increase of the BBB and cortical hemorrhages likely caused by impact-induced shear stress. Using TUNEL staining, frequent apoptotic cells were observed in the cortex underlying the impact although only rarely in the subcortical white matter and hippocampus. Animals showing prolonged apneas died and we arbitrarily chose a cut-off apnea length of 60s. Similarly, apnea immediately following impact is a feature of cFPI in the rat [37,38]. Since most animals had a substantially shorter apnea, a mean of 35 s, apnea-induced hypoxia contributing to our present results is unlikely.

Post-injury development of seizures is a feature of many experimental TBI models [39-41], although not previously evaluated in this mouse TAI model. We observed seizures immediately post-injury by direct visual inspection. In the lateral fluid percussion brain injury and weight drop injury in rats, there is immediate extracellular electrolyte and transmitter disturbances leading to cortical spreading depression and seizure activity [41,42]. In the original report using the cFPI model in mice [5], no TUNEL-positive cells, apnea or seizures were reported despite similar atmospheric pressure to the present report (1.7 *vs.* 1.5 atm, respectively). When using the FPI models, atmospheric pressure does not fully define injury severity in comparison between laboratories [29]. It is plausible that the apnea and seizures in our report reflect a more marked brain displacement also involving the brain stem and indicate a more severe injury level compared to the report by Greer *et al.*[5]. Additionally, the degree of cortical damage is associated with seizure susceptibility [43] and post-traumatic epilepsy is common in the lateral FPI model in mice [39]. Following cFPI in the rat, no enhanced seizure susceptibility to pentylenetetrazol (PTZ) was found [38], despite an increased neuronal excitability [44]. Although the detailed electrophysiological response to cFPI has not been established, immediate seizures have been observed in other diffuse head injury models [45] and it was thus not surprising to observe post-injury seizures also in the mouse cFPI model. Even though it may be argued that transient acute apnea and seizures add to the complexity of the cFPI model, these phenomena are important in a clinically-relevant model since they are commonly observed in TBI victims.

### Axonal injury after cFPI

Increased β-APP immunoreactivity was observed in the cortex, subcortical white matter, external capsule and the hippocampus of injured animals indicating that the cFPI model mimics human DAI. The immunoreactivity for β-APP in white matter tracts was transient and could not be observed beyond 3 days post-injury. Importantly, injured brainstem axons may play a major role in the induction of post-traumatic coma [46] and were also observed in the cFPI model. The β-APP staining was consistent with the spatial distribution of β-APP swellings previously observed in the rat model of mild cFPI [25,47-49]. In the first publication using the mouse cFPI model [5], swollen axons were observed in the neocortex and in the subcortical white matter already at 15 min post-injury. However, APP immunoreactivity was not observed beyond two days post-injury in the previous study [5], compared to three days in the present study. The mechanisms for the transient appearance of β-APP are unknown but may be due to accumulation of β-APP degrading enzymes within injured axons [50-52]. Increased β-APP immunostaining in white matter tracts is a consistent feature of experimental TBI models including the Marmarou impact acceleration and lateral fluid percussion injury (lFPI) models in rats [53-55]. Following TAI, secondary axotomy and subsequent Wallerian degeneration lead to downstream synaptic degeneration and deafferentation of postsynaptic targets [48,56]. However, perisomatic thalamic axotomy after cFPI in rats is associated with neuronal atrophy rather than cell death [49] and reactive sprouting accompanied by axonal elongation over time was observed following cFPI in mice [5]. These results suggest that endogenous restorative efforts are elicited post-injury and that treatments targeting axonal injury should be addressed in experimental TBI research.

### Marked astroglial cell reactivity after cFPI

Activation of astrocytes and reactive gliosis are key features observed in many CNS diseases including neurotrauma [57,58]. Reactive astrocytes play important roles in wound healing and neuronal regeneration and constitute the main cellular component of the glial scar [59]. Increased GFAP immunoreactivity is considered to be one sensitive marker of glial activation [60] and vimentin another early sign of astrocyte activation in CNS injury [61,62]. Following TBI, hypertrophic and proliferating astrocytes have been attributed to both beneficial and detrimental consequences. When reactive astrocytes were ablated following CCI in mice, there was an increased loss of cortical tissue suggesting that reactive astrocytes play an important role in preserving neural tissue and in restricting inflammation [63].

In our present study, vimentin immunoreactive cells were numerous in the cortex, in the subcortical white matter, in the hippocampus and in the internal capsule, while groups of GFAP positive cells were also observed in the thalamus. Both vimentin and GFAP immunoreactivity was slightly decreased at 7 days post-injury. In contrast to vimentin, the GFAP immunoreactivity was not increased in the internal capsule. These results indicate a different astroglial response between astrocytes expressing vimentin or GFAP [64,65]. Previously, a prolonged, widespread astrocytic response was observed in the subcortical white matter after CCI brain injury in mice and rats [66]. Vasogeni cedema, indicated by plasma protein leakage, was increased up to 7 days post-injury in the present study. The brain regions showing increased BBB permeability correlated with increased immunoreactivity for vimentin/GFAP and β-APP staining, similar to findings observed following CCI in the rat [62]. The exact function of vimentin in the adult brain following TBI is not known, although GFAP and vimentin may have overlapping functions since only mice with a gene deletion of both GFAP and vimentin exhibit attenuated glial scar reactions after neurotrauma [67].

These two astrocyte markers were thus up-regulated with, to some extent, different distribution and time course post-injury, in line with our previous findings of a wider distribution and longer time course of vimentin compared to GFAP following severe CCI mouse [62]. The combined results suggest variable astroglial activation depending on brain region and astrocyte subtype post-TBI. There is evidence that GFAP and vimentin expression is differentially regulated [61], and it is plausible that the delayed and more wide-spread vimentin expression observed in our present report is related to specific regional cytokine responses which should be addressed in future studies.

### The inflammatory response in white matter tracts after cFPI

Recently, it has become apparent that the central nervous system is not immunologically privileged. TBI induces an immune response, which includes both activation of microglia and infiltration of leukocytes and T-cells into injured brain tissue [68]. Our previous studies in a mouse CCI model showed a robust, early up-regulation of a number of inflammatory genes, including chemokines and their receptors, and a marked cellular inflammatory response consisting of infiltrating neutrophils and T-cells and activation of microglia [69-72]. Suppression of acute cytokine and chemokine up-regulation by anti-inflammatory agents has improved outcome in experimental TBI [73-75]. Recent clinical research also suggests that TBI activates the innate immune system in the brain in a complex interplay with the systemic immune system [76,77]. Importantly, recent data from DAI patients obtained using cerebral microdialysis demonstrate a complex temporal pattern of inflammatory biomarkers (*e.g.,* cytokines, chemokines), implying that inflammation may be an important mechanism in axonal injury [76]. In a previous study in CCI mice both microglia and invading monocytes/macrophages were identified by flow cytometry as a part of the post-traumatic inflammatory response [70]. In our present study, activated microglia/macrophages stained with MAC-2 remained high in the subcortical white matter from one to seven days post-injury. This antibody does not distinguish between macrophages and microglial cell types and morphology alone may not be sufficient to differentiate between these two cell types. An extended study using additional antibody markers following TBI could provide information on the distinction between resident microglia and blood-stream derived macrophages.

We also observed a limited number of neutrophils infiltrating the white matter tracts, peaking at 1 day after cFPI. Similarly, the infiltration of CD3+ T-cells in the white matter tracts peaked at day 1 post-injury and declined at the later time points. In summary, there was a robust inflammatory response after cFPI in mice, especially at 1 day post-injury. Microglia is one cell type that synthesizes and releases interleukin-1β (IL-1β), a pro-inflammatory cytokine protein which is an important mediator of the inflammatory response [78-81]. In a previous report, microglial activation was observed after cFPI in the rat as early as 6 hours post-injury and there was a spatiotemporal relationship with TAI [25]. Rapid recruitment of neutrophils is part of the early inflammatory response following human TBI [82]. Neutrophils may trigger increased vascular permeability [83,84] and release of reactive oxygen species, proteases and pro-inflammatory cytokines [85]. Further, neutrophil depletion at the time of TBI was neuroprotective [86]. Although T-lymphocytes have been shown to infiltrate the brain parenchyma in focal TBI models in the rat [71,87], only a minimal cortical infiltration of T-cell receptor (TcR)-positive cells was observed at seven days following CCI in the mouse [80]. Here, we used the marker CD3, required for T-cell activation, of which antigen is bound to the membrane of all mature T-cells. The CD3 immunoreactivity peaked at 1 day post-injury, and the number of T-cells in the white matter tract was significantly increased. However, further analysis is needed to distinguish between different T-lymphocyte subtypes in this TBI model.

### Complex behavioral changes are elicited by cFPI

Functional outcome analysis is crucial in TBI research and we hypothesized that cFPI model could induce behavioral disturbances. In the first trial in the MCSF, all animals were facing a novel environment and they freely started to explore the arena using various explorative strategies [28,88,89]. Compared to sham-injured animals, the behavioral pattern of cFPI animals were different. In both trials, cFPI mice were passing the centre of the arena fewer times, hence changing zones in the arena, compared to sham-injured controls. Furthermore, the cFPI mice had less activity in the whole arena, made fewer visits to corridors and spent more time running around in circles in the centre of the arena indicating a pathological behavior.

Although the cFPI model and other models of TAI in the rat induce cognitive impairment [90], there are no previous reports evaluating complex behavioral disturbances in rodent TAI models. To test the hypothesis that the behavioral disturbances induced by cFPI were unique, we extracted previously published behavioral MCSF data [28] from mice subjected to the focal CCI model. From the PCA, it was apparent that mice subjected to a focal TBI (CCI) or cFPI used different behavioral strategies and displayed altered behavioral patterns (Figures 8 and 9). In the first trial, cFPI-animals spent more time in the centre of the arena and made more rears. In the second trial, cFPI mice remained more active, spent more time in the central circle, made more visits to the centre of the arena and made more rears compared to the CCI-injured mice [28]. Although direct comparisons between the previously published [28] and present data must be made with caution, an identical experimental set-up was used and all behavioral testing was performed by the same investigator. These results argue that the cFPI model induces unique behavioral deficits with a markedly different pattern compared to focal TBI.

In our report, the first to study behavioral outcome in the cFPI model in mice, it was obvious that the cFPI model induced a unique pathological behavior pattern different from that observed in sham-injured controls and in mice subjected to focal TBI [28]. Our results support the hypothesis that axonal injury is a key contributor to complex behavioral disturbances post-TBI. In the development of novel TAI treatment options, behavioral outcome studies are crucial and the MCSF appears to be a suitable test for addressing complex behavioral changes induced by axonal injury.

## Conclusions

The cFPI model in mice resulted in increased blood–brain barrier permeability, diffuse axonal injury, widespread astroglial cell reactivity and a robust inflammatory response including activation of microglia/macrophages, infiltration of neutrophils and T-cells. The distribution of axonal damage was less widespread than the immunoreactivity of glial cells positive for vimentin and GFAP. The inflammatory response was restricted to the cortex and the subcortical white matter and included areas of axonal injury. Additionally, there were complex behavioral changes observed in brain-injured animals that appeared unique to this TBI model. Our results argue that behavioral evaluation is crucial in the evaluation of traumatic axonal injury. The widespread axonal injury observed in this model reflects many of the clinical features observed in TBI patients. Further analysis including evaluation at later time-points is needed to study the long-term consequences initiated by the cFPI. It is likely that the mouse central fluid percussion injury model can be an important complement to other TBI models for the study of pathological mechanisms including neuroinflammation and treatment strategies for diffuse traumatic axonal injury.

## Abbreviations

BBB: Blood brain barrier; β-APP: β-amyloid precursor protein; CCI: Controlled cortical impact; cFPI: Central fluid percussion injury; CNS: Central nervous system; DAI: Diffuse axonal injury; MCSF: Multivariate concentric square field test; GFAP: Glial fibrillary acidic protein; PCA: Principal component analysis; ROS: Reactive oxygen species; S-PBN: 2-sulfophenyl-N-tert-butyl nitrone; TAI: Traumatic axonal injury; TBI: Traumatic brain injury; TcR: T-cell receptor; TUNEL: Terminal deoxynucleotidyltransferase dUTP nick end labeling.

## Competing interests

The authors declare that there are no competing interests.

## Author’s contributions

S E-L performed experiments, analyzed data and contributed to writing the manuscript. JF and OK performed experiments and analyzed data. BJ.M, AL, LH and NM participated in the design and coordination of the study and in writing the manuscript. All authors read and approved the final manuscript.

## References

[B1] Gennarelli TA Thibault LE Adams JH Graham DI Thompson CJ Marcincin RP Diffuse axonal injury and traumatic coma in the primate Ann Neurol 1982 12 564 574 10.1002/ana.410120611 7159060

[B2] Adams JH Doyle D Ford I Gennarelli TA Graham DI McLellan DR Diffuse axonal injury in head injury: definition, diagnosis and grading Histopathology 1989 15 49 59 10.1111/j.1365-2559.1989.tb03040.x 2767623

[B3] Abou-Hamden A Blumbergs PC Scott G Manavis J Wainwright H Jones N McLean J Axonal injury in falls J Neurotrauma 1997 14 699 713 10.1089/neu.1997.14.699 9383089

[B4] Pittella JE Gusmao SN The conformation of the brain plays an important role in the distribution of diffuse axonal injury in fatal road traffic accident Arq Neuropsiquiatr 2004 62 406 412 10.1590/S0004-282X2004000300007 15273836

[B5] Greer JE McGinn MJ Povlishock JT Diffuse traumatic axonal injury in the mouse induces atrophy, c-Jun activation, and axonal outgrowth in the axotomized neuronal population J Neurosci 2011 31 5089 5105 10.1523/JNEUROSCI.5103-10.2011 21451046 PMC3076099

[B6] Strich S Diffuse degeneration of the cerebral white matter in severe dementia following head injury J Neurol Neurosurg Psychiatry 1956 19 163 185 10.1136/jnnp.19.3.163 13357957 PMC497203

[B7] Povlishock JT Katz DI Update of neuropathology and neurological recovery after traumatic brain injury J Head Trauma Rehabil 2005 20 76 94 10.1097/00001199-200501000-00008 15668572

[B8] Graham DI McLellan D Adams JH Doyle D Kerr A Murray LS The neuropathology of the vegetative state and severe disability after non-missile head injury Acta NeurochirSuppl (Wien) 1983 32 65 67 10.1007/978-3-7091-4147-2_6 6581706

[B9] Adams JH Jennett B McLellan DR Murray LS Graham DI The neuropathology of the vegetative state after head injury J Clin Pathol 1999 52 804 806 10.1136/jcp.52.11.804 10690167 PMC501589

[B10] Lipton ML Gellella E Lo C Gold T Ardekani BA Shifteh K Bello JA Branch CA Multifocal white matter ultrastructural abnormalities in mild traumatic brain injury with cognitive disability: a voxel-wise analysis of diffusion tensor imaging J Neurotrauma 2008 25 1335 1342 10.1089/neu.2008.0547 19061376

[B11] Lipton ML Gulko E Zimmerman ME Friedman BW Kim M Gellella E Gold T Shifteh K Ardekani BA Branch CA Diffusion-tensor imaging implicates prefrontal axonal injury in executive function impairment following very mild traumatic brain injury Radiology 2009 252 816 824 10.1148/radiol.2523081584 19567646

[B12] Medana IM Esiri MM Axonal damage: a key predictor of outcome in human CNS diseases Brain 2003 126 515 530 10.1093/brain/awg061 12566274

[B13] Huebner EA Strittmatter SM Axon regeneration in the peripheral and central nervous systems Results Probl Cell Differ 2009 48 339 351 19582408 10.1007/400_2009_19 PMC2846285

[B14] Vink R Van Den Heuvel C Recent advances in the development of multifactorial therapies for the treatment of traumatic brain injury Expert Opin Investig Drugs 2004 13 1263 1274 10.1517/13543784.13.10.1263 15461556

[B15] Marklund N Bakshi A Castelbuono D McIntosh T Evaluation of pharmacological treatment strategies in traumatic brain injury Curr Pharm Des 2006 12 13 1645 1680 10.2174/138161206776843340 16729876

[B16] Gentleman SM Nash MJ Sweeting CJ Graham DI Roberts GW ß-Amyloid precursor protein (β-APP) as a marker for axonal injury after head injury Neurosci Lett 1993 160 139 144 10.1016/0304-3940(93)90398-5 8247344

[B17] Sherriff FE Bridges LR Sivaloganathan S Early detection of axonal injury after human head trauma using immunocytochemistry for beta-amyloid precursor protein Acta Neuropathol 1994 87 55 62 10.1007/BF00386254 8140894

[B18] Blumbergs PC Scott G Manavis J Wainwright H Simpson DA Topography of axonal injury as defined by amyloid precursor protein and the sector scoring method in mild and severe closed head injury J Neurotrauma 1995 12 565 572 10.1089/neu.1995.12.565 8683607

[B19] McKenzie KJ McLellan DR Gentleman SM Maxwell WL Gennarelli TA Graham DI Is beta-APP a marker of axonal damage in short-surviving head injury? Acta Neuropathol 1996 92 608 613 10.1007/s004010050568 8960319

[B20] Geddes JF Vowles GH Beer TW Ellison DW The diagnosis of diffuse axonal injury: implications for forensic practice Neuropathol Appl Neurobiol 1997 23 339 347 10.1111/j.1365-2990.1997.tb01305.x 9292874

[B21] Lewen A Li GL Nilsson P Olsson Y Hillered L Traumatic brain injury in rat produces changes of beta-amyloid precursor protein immunoreactivity Neuroreport 1995 6 357 360 10.1097/00001756-199501000-00032 7756628

[B22] Johnson VE Stewart W Smith DH Axonal pathology in traumatic brain injury Exp Neurol 2012 Epub ahead of print 10.1016/j.expneurol.2012.01.013 PMC3979341 22285252

[B23] Dixon CE Lyeth BG Povlishock JT Findling RL Hamm RJ Marmarou A Young HF Hayes RL A fluid percussion model of experimental brain injury in the rat J Neurosurg 1987 67 110 119 10.3171/jns.1987.67.1.0110 3598659

[B24] Marmarou A Foda MA van den Brink W Campbell JHK Demetriadou K A new model of diffuse brain injury in rats. Part I: Pathophysiology and biomechanics J Neurosurg 1994 80 2 291 300 10.3171/jns.1994.80.2.0291 8283269

[B25] Kelley BJ Lifshitz J Povlishock JT Neuroinflammatory responses after experimental diffuse traumatic brain injury J Neuropathol Exp Neurol 2007 66 989 1001 10.1097/NEN.0b013e3181588245 17984681

[B26] Venkatesan C Chrzaszcz M Choi N Wainwright MS Chronic upregulation of activated microglia immunoreactive for galectin-3/Mac-2 and nerve growth factor following diffuse axonal injury J Neuroinflammation 2010 7 32 10.1186/1742-2094-7-32 20507613 PMC2891720

[B27] Dixon CE Clifton GL Lighthall JW Yaghmai AA Hayes RL A controlled cortical impact model of traumatic brain injury in the rat J Neurosci Methods 1991 39 253 262 10.1016/0165-0270(91)90104-8 1787745

[B28] Ekmark-Lewen S Lewen A Meyerson BJ Hillered L The multivariate concentric square field test reveals behavioral profiles of risk taking, exploration, and cognitive impairment in mice subjected to traumatic brain injury J Neurotrauma 2010 27 1643 1655 10.1089/neu.2009.0953 20578827

[B29] Thompson HJ Lifshitz J Marklund N Grady MS Graham DI Hovda DA McIntosh TK Lateral fluid percussion brain injury: a 15-year review and evaluation J Neurotrauma 2005 22 42 75 10.1089/neu.2005.22.42 15665602

[B30] Marklund N Bareyre FM Royo NC Thompson HJ Mir AK Grady MS Schwab ME McIntosh TK Cognitive outcome following brain injury and treatment with an inhibitor of Nogo-A in association with an attenuated downregulation of hippocampal growth-associated protein-43 expression J Neurosurg 2007 107 844 853 10.3171/JNS-07/10/0844 17937233 PMC2366808

[B31] PaxinosGaF KBJ The Mouse Brain in Stereotaxic Coordinates 2001 2 New York: Academic Press

[B32] Eriksson LJE Kettaneh-Wold N Trygg J Wikström C Wold S Multi- and megavariate data analysis. Part I: basic principles and applications.Umeå 2006 Sweden: Umetrics AB

[B33] Eriksson L Antti H Gottfries J Holmes E Johansson E Lindgren F Long I Lundstedt T Trygg J Wold S Using chemometrics for navigating in the large data sets of genomics, proteomics, and metabonomics (gpm) Anal Bioanal Chem 2004 380 419 429 10.1007/s00216-004-2783-y 15448969

[B34] Eriksson L Andersson PL Johansson E Tysklind M Megavariate analysis of environmental QSAR data. Part II–investigating very complex problem formulations using hierarchical, non-linear and batch-wise extensions of PCA and PLS Mol Divers 2006 10 187 205 10.1007/s11030-006-9026-4 16802062

[B35] Lewen A Fredriksson A Li GL Olsson Y Hillered L Behavioural and morphological outcome of mild cortical contusion trauma of the rat brain: influence of NMDA-receptor blockade Acta Neurochir (Wien) 1999 141 193 202 10.1007/s007010050286 10189503

[B36] Cole JT Yarnell A Kean WS Gold E Lewis B Ren M McMullen DC Jacobowitz DM Pollard HB O'Neill JT Grunberg NE Dalgard CL Frank JA Watson WD Craniotomy: true sham for traumatic brain injury, or a sham of a sham? J Neurotrauma 2011 28 359 369 10.1089/neu.2010.1427 21190398 PMC3057208

[B37] Stone JR Singleton RH Povlishock JT Intra-axonal neurofilament compaction does not evoke local axonal swelling in all traumatically injured axons Exp Neurol 2001 172 320 331 10.1006/exnr.2001.7818 11716556

[B38] Hamm RJ Pike BR Temple MD O'Dell DM Lyeth BG The effect of postinjury kindled seizures on cognitive performance of traumatically brain-injured rats Exp Neurol 1995 136 143 148 10.1006/exnr.1995.1091 7498404

[B39] Bolkvadze T Pitkanen A Development of post-traumatic epilepsy after controlled cortical impact and lateral fluid-percussion-induced brain injury in the mouse J Neurotrauma 2012 29 789 812 10.1089/neu.2011.1954 22023672

[B40] Golarai G Greenwood AC Feeney DM Connor JA Physiological and structural evidence for hippocampal involvement in persistent seizure susceptibility after traumatic brain injury J Neurosci 2001 21 8523 8537 11606641 10.1523/JNEUROSCI.21-21-08523.2001 PMC6762822

[B41] Nilsson P Ronne-Engstrom E Flink R Ungerstedt U Carlson H Hillered L Epileptic seizure activity in the acute phase following cortical impact trauma in rat Brain Res 1994 637 227 232 10.1016/0006-8993(94)91237-8 8180800

[B42] Rogatsky GG Sonn J Kamenir Y Zarchin N Mayevsky A Relationship between intracranial pressure and cortical spreading depression following fluid percussion brain injury in rats J Neurotrauma 2003 20 1315 1325 10.1089/089771503322686111 14748980

[B43] Kharatishvili I Pitkanen A Association of the severity of cortical damage with the occurrence of spontaneous seizures and hyperexcitability in an animal model of posttraumatic epilepsy Epilepsy Res 2010 90 1–2 47 59 20435440 10.1016/j.eplepsyres.2010.03.007

[B44] Reeves TM Lyeth BG Povlishock JT Long-term potentiation deficits and excitability changes following traumatic brain injury Exp Brain Res 1995 106 248 256 8566189 10.1007/BF00241120

[B45] Nilsson B Ponten U Voigt G Experimental head injury in the rat. Part 1: Mechanics, pathophysiology, and morphology in an impact acceleration trauma model J Neurosurg 1977 47 241 251 10.3171/jns.1977.47.2.0241 874547

[B46] Smith DH Nonaka M Miller R Leoni M Chen XH Alsop D Meaney DF Immediate coma following inertial brain injury dependent on axonal damage in the brainstem J Neurosurg 2000 93 315 322 10.3171/jns.2000.93.2.0315 10930019

[B47] Singleton RH Zhu J Stone JR Povlishock JT Traumatically induced axotomy adjacent to the soma does not result in acute neuronal death J Neurosci 2002 22 791 802 11826109 10.1523/JNEUROSCI.22-03-00791.2002 PMC6758486

[B48] Kelley BJ Farkas O Lifshitz J Povlishock JT Traumatic axonal injury in the perisomatic domain triggers ultrarapid secondary axotomy and Wallerian degeneration Exp Neurol 2006 198 350 360 10.1016/j.expneurol.2005.12.017 16448652

[B49] Lifshitz J Kelley BJ Povlishock JT Perisomatic thalamic axotomy after diffuse traumatic brain injury is associated with atrophy rather than cell death J Neuropathol Exp Neurol 2007 66 218 229 10.1097/01.jnen.0000248558.75950.4d 17356383

[B50] Uryu K Chen XH Martinez D Browne KD Johnson VE Graham DI Lee VM Trojanowski JQ Smith DH Multiple proteins implicated in neurodegenerative diseases accumulate in axons after brain trauma in humans Exp Neurol 2007 208 185 192 10.1016/j.expneurol.2007.06.018 17826768 PMC3979356

[B51] Chen XH Johnson VE Uryu K Trojanowski JQ Smith DH A lack of amyloid beta plaques despite persistent accumulation of amyloid beta in axons of long-term survivors of traumatic brain injury Brain Pathol 2009 19 214 223 10.1111/j.1750-3639.2008.00176.x 18492093 PMC3014260

[B52] Johnson VE Stewart W Smith DH Traumatic brain injury and amyloid-beta pathology: a link to Alzheimer's disease? Nat Rev Neurosci 2010 11 361 370 20216546 10.1038/nrn2808 PMC3979339

[B53] Li Y Zhang L Kallakuri S Zhou R Cavanaugh JM Quantitative relationship between axonal injury and mechanical response in a rodent head impact acceleration model J Neurotrauma 2011 28 1767 1782 10.1089/neu.2010.1687 21895482 PMC3172873

[B54] Bramlett HM Kraydieh S Green EJ Dietrich WD Temporal and regional patterns of axonal damage following traumatic brain injury: a beta-amyloid precursor protein immunocytochemical study in rats J Neuropathol Exp Neurol 1997 56 10 1132 1141 10.1097/00005072-199710000-00007 9329457

[B55] Pierce JE Trojanowski JQ Graham DI Smith DH McIntosh TK Immunohistochemical characterization of alterations in the distribution of amyloid precursor proteins and beta-amyloid peptide after experimental brain injury in the rat J Neurosci 1996 16 3 1083 1090 8558237 10.1523/JNEUROSCI.16-03-01083.1996 PMC6578806

[B56] Rafols JA Morgan R Kallakuri S Kreipke CW Extent of nerve cell injury in Marmarou's model compared to other brain trauma models Neurol Res 2007 29 348 355 10.1179/016164107X204657 17626729

[B57] Kimelberg HK Norenberg MD Salzman SK, Faden AI Astrocytic Responses to Central Nervous System Trauma The Neurobiology of Central Nervous System Trauma 1994 New York: Oxford University Press 193 208

[B58] Norenberg MD Astrocyte response to CNS Injury J Neuropath ExpNeur 1994 53 213 220 10.1097/00005072-199405000-00001 8176405

[B59] Stichel CC Muller HW The CNS lesion scar: new vistas on an old regeneration barrier Cell Tissue Res 1998 294 1 9 10.1007/s004410051151 9724451

[B60] Bignami A Glial cells in the central nervous system Discussions in Neuroscience 1991 111 9 44

[B61] Calvo JL Carbonell AL Boya J Co-expression of glial fibrillary acidic protein and vimentin in reactive astrocytes following brain injury in rats Brain Res 1991 566 333 336 10.1016/0006-8993(91)91720-L 1814551

[B62] Schiffer D Giordana MT Migheli A Giaccone G Pezzotta S Mauro A Glial fibrillary acidic protein and vimentin in the experimental glial reaction of the rat brain Brain Res 1986 374 110 118 10.1016/0006-8993(86)90399-9 2424556

[B63] Myer DJ Gurkoff GG Lee SM Hovda DA Sofroniew MV Essential protective roles of reactive astrocytes in traumatic brain injury Brain 2006 129 2761 2772 10.1093/brain/awl165 16825202

[B64] Ridet JL Malhotra SK Privat A Gage FH Reactive astrocytes: cellular and molecular cues to biological function Trends Neurosci 1997 20 570 577 10.1016/S0166-2236(97)01139-9 9416670

[B65] Pixley SK de Vellis J Transition between immature radial glia and mature astrocytes studied with a monoclonal antibody to vimentin Brain Res 1984 317 201 209 6383523 10.1016/0165-3806(84)90097-x

[B66] Ekmark-Lewen S Lewen A Israelsson C Li GL Farooque M Olsson Y Ebendal T Hillered L Vimentin and GFAP responses in astrocytes after contusion trauma to the murine brain Restor Neurol Neurosci 2010 28 311 321 20479526 10.3233/RNN-2010-0529

[B67] Pekny M Eliasson C Siushansian R Ding M Dixon SJ Pekna M Wilson JX Hamberger A The impact of genetic removal of GFAP and/or vimentin on glutamine levels and transport of glucose and ascorbate in astrocytes Neurochem Res 1999 24 1357 1362 10.1023/A:1022572304626 10555775

[B68] Morganti-Kossmann MC Satgunaseelan L Bye N Kossmann T Modulation of immune response by head injury Injury 2007 38 1392 1400 10.1016/j.injury.2007.10.005 18048036

[B69] Israelsson C Bengtsson H Kylberg A Kullander K Lewen A Hillered L Ebendal T Distinct cellular patterns of upregulated chemokine expression supporting a prominent inflammatory role in traumatic brain injury J Neurotrauma 2008 25 959 974 10.1089/neu.2008.0562 18665806

[B70] Israelsson C Bengtsson H Lobell A Nilsson LN Kylberg A Isaksson M Wootz H Lannfelt L Kullander K Hillered L Ebendal T Appearance of Cxcl10-expressing cell clusters is common for traumatic brain injury and neurodegenerative disorders Eur J Neurosci 2010 31 852 863 10.1111/j.1460-9568.2010.07105.x 20374285

[B71] Clausen F Lorant T Lewen A Hillered L T lymphocyte trafficking: a novel target for neuroprotection in traumatic brain injury J Neurotrauma 2007 24 1295 1307 10.1089/neu.2006.0258 17711391

[B72] Clausen F Hanell A Israelsson C Hedin J Ebendal T Mir AK Gram H Marklund N Neutralization of interleukin-1beta reduces cerebral edema and tissue loss and improves late cognitive outcome following traumatic brain injury in mice Eur J Neurosci 2011 34 110 123 10.1111/j.1460-9568.2011.07723.x 21623956

[B73] Lloyd E Somera-Molina K Van Eldik LJ Watterson DM Wainwright MS Suppression of acute proinflammatory cytokine and chemokine upregulation by post-injury administration of a novel small molecule improves long-term neurologic outcome in a mouse model of traumatic brain injury J Neuroinflammation 2008 5 28 10.1186/1742-2094-5-28 18590543 PMC2483713

[B74] Yatsiv I Grigoriadis N Simeonidou C Stahel PF Schmidt OI Alexandrovitch AG Tsenter J Shohami E Erythropoietin is neuroprotective, improves functional recovery, and reduces neuronal apoptosis and inflammation in a rodent model of experimental closed head injury FASEB J 2005 19 1701 1703 16099948 10.1096/fj.05-3907fje

[B75] Bye N Habgood MD Callaway JK Malakooti N Potter A Kossmann T Morganti-Kossmann MC Transient neuroprotection by minocycline following traumatic brain injury is associated with attenuated microglial activation but no changes in cell apoptosis or neutrophil infiltration Exp Neurol 2007 204 220 233 10.1016/j.expneurol.2006.10.013 17188268

[B76] Helmy A Carpenter KL Menon DK Pickard JD Hutchinson PJ The cytokine response to human traumatic brain injury: temporal profiles and evidence for cerebral parenchymal production J Cereb Blood Flow Metab 2011 31 658 670 10.1038/jcbfm.2010.142 20717122 PMC3049520

[B77] Helmy A De Simoni MG Guilfoyle MR Carpenter KL Hutchinson PJ Cytokines and innate inflammation in the pathogenesis of human traumatic brain injury Prog Neurobiol 2011 95 352 372 10.1016/j.pneurobio.2011.09.003 21939729

[B78] Giulian D Baker TJ Shih LC Lachman LB Interleukin 1 of the central nervous system is produced by ameboid microglia J Exp Med 1986 164 594 604 10.1084/jem.164.2.594 3487617 PMC2188228

[B79] Pearson VL Rothwell NJ Toulmond S Excitotoxic brain damage in the rat induces interleukin-1beta protein in microglia and astrocytes: correlation with the progression of cell death Glia 1999 25 311 323 10.1002/(SICI)1098-1136(19990215)25:4<311::AID-GLIA1>3.0.CO;2-E 10028914

[B80] Clausen F Hanell A Bjork M Hillered L Mir AK Gram H Marklund N Neutralization of interleukin-1beta modifies the inflammatory response and improves histological and cognitive outcome following traumatic brain injury in mice Eur J Neurosci 2009 30 385 396 10.1111/j.1460-9568.2009.06820.x 19614750

[B81] Rivest S Regulation of innate immune responses in the brain Nat Rev Immunol 2009 9 429 439 10.1038/nri2565 19461673

[B82] Holmin S Soderlund J Biberfeld P Mathiesen T Intracerebral inflammation after human brain contusion Neurosurgery 1998 42 291 298 Discussion 298–299 10.1097/00006123-199802000-00047 9482179

[B83] Wedmore CV Williams TJ Control of vascular permeability by polymorphonuclear leukocytes in inflammation Nature 1981 289 646 650 10.1038/289646a0 7464931

[B84] Lindbom L Regulation of vascular permeability by neutrophils in acute inflammation Chem Immunol Allergy 2003 83 146 166 12947983 10.1159/000071559

[B85] Jin R Yang G Li G Inflammatory mechanisms in ischemic stroke: role of inflammatory cells J Leukoc Biol 2010 87 779 789 10.1189/jlb.1109766 20130219 PMC2858674

[B86] Kenne E Erlandsson A Lindbom L Hillered L Clausen F Neutrophil depletion reduces edema formation and tissue loss following traumatic brain injury in mice J Neuroinflammation 2012 9 17 10.1186/1742-2094-9-17 22269349 PMC3292978

[B87] Holmin S Mathiesen T Shetye J Biberfeld P Intracerebral inflammatory response to experimental brain contusion Acta Neurochir (Wien) 1995 132 1–3 110 119 7754844 10.1007/BF01404857

[B88] Roman E Gustafsson L Berg M Nylander I Behavioral profiles and stress-induced corticosteroid secretion in male Wistar rats subjected to short and prolonged periods of maternal separation Horm Behav 2006 50 736 747 10.1016/j.yhbeh.2006.06.016 16876800

[B89] Meyerson BJ Augustsson H Berg M Roman E The Concentric Square Field: a multivariate test arena for analysis of explorative strategies Behav Brain Res 2006 168 100 113 10.1016/j.bbr.2005.10.020 16356558

[B90] Maruichi K Kuroda S Chiba Y Hokari M Shichinohe H Hida K Iwasaki Y Transplanted bone marrow stromal cells improves cognitive dysfunction due to diffuse axonal injury in rats Neuropathology 2009 29 422 432 10.1111/j.1440-1789.2008.00995.x 19170895

